# Targeting of erbB3 receptor to overcome resistance in cancer treatment

**DOI:** 10.1186/1476-4598-13-105

**Published:** 2014-05-08

**Authors:** Jian Ma, Hui Lyu, Jingcao Huang, Bolin Liu

**Affiliations:** 1 Department of Pathology, School of Medicine, University of Colorado Anschutz Medical Campus, Aurora, CO, USA; 2 International Medical Centre of PLA General Hospital, Beijing, China

**Keywords:** erbB3, Cell signaling, Drug resistance, Targeted therapy, Cancer

## Abstract

The erbB receptors, including the epidermal growth factor receptor (EGFR), erbB2 (also known as HER2/neu), erbB3 (or HER3), and erbB4 (or HER4), are often aberrantly activated in a wide variety of human cancers. They are excellent targets for selective anti-cancer therapies because of their transmembrane location and pro-oncogenic activity. While several therapeutic agents against erbB2 and/or EGFR have been used in the treatment of human cancers with efficacy, there has been relatively less emphasis on erbB3 as a molecular target. Elevated expression of erbB3 is frequently observed in various malignancies, where it promotes tumor progression via interactions with other receptor tyrosine kinases (RTKs) due to its lack of or weak intrinsic kinase activity. Studies on the underlying mechanisms implicate erbB3 as a major cause of treatment failure in cancer therapy, mainly through activation of the PI-3 K/Akt, MEK/MAPK, and Jak/Stat signaling pathways as well as Src kinase. It is believed that inhibition of erbB3 signaling may be required to overcome therapeutic resistance and effectively treat cancers. To date, no erbB3-targeted therapy has been approved for cancer treatment. Targeting of erbB3 receptor with a monoclonal antibody (Ab) is the only strategy currently under preclinical study and clinical evaluation. In this review, we focus on the role of erbB3-initiated signaling in the development of cancer drug resistance and discuss the latest advances in identifying therapeutic strategies inactivating erbB3 to overcome the resistance and enhance efficacy of cancer therapeutics.

## Introduction

ErbB3 (HER3) belongs to the erbB (HER) receptor family of type I receptor tyrosine kinases (RTKs). The erbB receptor family consists of the epidermal growth factor receptor (EGFR), erbB2 (HER2/neu), erbB3 (HER3), and erbB4 (HER4). It is arguably one of the most important receptor families in the context of development and tumorigenesis [1,2]. Abnormal expression and/or aberrant activation of the erbB receptors have been demonstrated in a wide variety of human cancers [3]. Dimerization of erbB receptors is an essential step for their function and activation of the downstream signaling pathways [3,4]. ErbB receptors normally exist as inactive monomers, with molecular folding to prevent dimerization, with the exception of erbB2 [5,6]. As a consequence of erbB receptor dimerization, the intracellular tyrosine kinase activity is activated and the tyrosine residues on the C-terminal tails are phosphorylated. This subsequently leads to the recruitment of adaptor proteins and activation of downstream signaling pathways. These induce diverse biologic responses, including cell proliferation, maturation, survival, apoptosis, and angiogenesis [3,7]. There are unique features of erbB2 and erbB3 receptors as compared to other family members. The binding of erbB3 ligand (heregulin, HRG) exposes a dimerization arm in the extracellular domain of erbB3 and promotes receptor-receptor interactions [8]. ErbB3 has been considered as a “kinase-dead” receptor [9,10], because it lacks significant intrinsic kinase activity [11]. Thus, in order for erbB3 to induce cell signaling, it must be phosphorylated by its interactive partners, of these, erbB2 is the most important one [12]. In contrast, the erbB2 receptor has tyrosine kinase activity, but it has no known ligand. ErbB2 exists in a constitutively active conformation with an exposed dimerization arm [5], that makes it as the preferred dimerization partner for other erbB receptors [13].

ErbB3 is frequently co-expressed with other RTKs in cancer cells. It promotes tumor initiation and progression mainly through activation of the oncogenic PI-3 K/Akt signaling. The erbB3/PI-3 K/Akt pathway is a major cause of treatment failure in cancer therapy because of its role in therapeutic resistance [14]. It also plays an important role in the development of various human cancers, including erbB2-overexpressing (erbB2+) breast cancer [15,16], castration-resistant prostate cancer (CRPC) [17], platinum-resistant/refractory ovarian cancer [18,19], and EGFR tyrosine kinase inhibitor (TKI)-resistant non-small cell lung cancer (NSCLC) [20,21]. For these cancers in particular, erbB3 inhibition may be required to effectively eradicate cancerous cells. Targeting of erbB3 with a blocking antibody (Ab) is the only strategy currently being investigated in preclinical [22,23] and clinical studies (http://www.clinicaltrials.gov), because it lacks appreciable kinase activity [10,11]. Several anti-erbB3 monoclonal Abs that prevent ligand-induced activation of erbB3, such as MM-121 and MM-111 (Merrimack Pharmaceuticals, Cambridge, MA) and U3-1287/AMG 888 (Amgen Inc., Thousand Oaks, CA) have shown significant antitumor activity *in vitro* and *in vivo*[22-25]. This review summarizes the latest advances in our understanding of the role of erbB3 signaling in cancer development and discusses novel strategies inactivating erbB3 to enhance the efficacy of cancer therapeutics.

### Characteristics of erbB3 receptor and its role in tumorigenesis

ErbB3 receptors are not capable of forming homo-dimers, but can induce activation of multiple downstream signaling pathways, such as PI-3 K/Akt, MEK/MAPK, PLCγ/PKC, Jak/Stat, and Src kinase, via hetero-dimerization with another RTK [10,26]. Studies of comparing sequences of all the erbB receptors reveal that the tyrosine kinase domain of erbB3 diminishes several key residues, which may explain the lack of, or much lower, intrinsic kinase activity in erbB3 [10,11]. However, once its ligand, heregulin (HRG) (also called neuregulin (NRG)) binds to erbB3, it can recruit another erbB receptor to form hetero-dimers, which leads to activation of the erbB3 receptor. ErbB3 is a more potent partner than other family members for the oncogenic activity of erbB2 [16,27-29]. It has been reported that erbB3 functions primarily to drive erbB2-mediated cell signaling [16,30]. Our recent studies using both mouse and human mammary/breast cancer cell models indicate that the existence of erbB3 is required to maintain erbB2’s tyrosine kinase activity [31,32].

Amplification and/or overexpression of *erbB3* are frequently observed in various malignancies, such as cancers of breast, gastric, ovarian, prostate, and bladder, colorectal carcinoma, squamous cell carcinoma of the head and neck, and melanoma [16,33,34]. A recent report identified somatic mutations of *erbB3* occurring in approximately 11% of colon and gastric cancers [35]. Similar to wild type erbB3, the oncogenic activity of mutant erbB3 also depends upon the kinase-active erbB2. The erbB3 mutants transform colonic and breast epithelial cells in a ligand-independent manner [35]. In breast cancer, both mRNA expression and protein levels of *erbB3* are upregulated. Most metastatic breast cancers show expression of either EGFR or erbB2, whereas upregulation of both is not typical [36]. In contrast, co-expression of erbB2 and erbB3 is a common event in breast cancers [37] and breast cancer-derived cell lines [38]. We and others have reported that overexpression of endogenous mouse erbB3, and its association with the transgene encoded erbB2, promotes mammary tumorigenesis in the *erbB2*/*neu*-transgenic mice [39,40]. ErbB3 serves as a critical co-receptor of erbB2, and its expression is a rate-limiting factor for erbB2-induced breast cancer cell survival and proliferation [15,16]. In erbB2+ breast cancer tissues, preferential phosphorylation of erbB3, but not EGFR, has been observed [16]. ErbB3 might also be the preferred dimerization partner for EGFR in melanoma and pancreatic cancer [41,42].

Elevated expression of erbB3 protein has been reported in 50-70% of human breast cancers [43-45], and it seems to be associated with tumor size, metastasis, and recurrence [46,47]. However, the prognostic value of erbB3 expression in breast cancer has been controversial [45-48]. Some studies show that erbB3 expression significantly reduces the overall survival and disease-free survival of breast cancer patients [37,49,50]. Others report a positive prognostic significance for erbB3 expression [48,51,52]. A number of hypotheses have been proposed to explain the dichotomous findings. They include: 1) a naturally occurring secreted isoform of erbB3 (p85-soluble erbB3) which can bind to the ligand HRG with high affinity, thereby blocks HRG binding to the full length erbB3 on cell surface and inhibits erbB3 activation [53]. This observation could be associated with the positive prognostic value of erbB3 in some studies. 2) subcellular distribution of erbB3 receptors may influence their biological activity. While erbB3 pool is mainly within the intracellular compartments, it seems that the levels of phosphorylated erbB3 (P-erbB3) and its activity are associated with erbB3 re-localization to the plasma membrane [54]. 3) The ligands such as HRGs may affect the distribution of erbB3 and increase the membrane levels of the receptor [54]. Thus, when we evaluate the impact of erbB3 on clinical outcome of breast cancer patients, it would be better to consider not only its expression and interactions with other RTKs like erbB2, but also its subcellular distribution as well as the expression levels of HRG. Nonetheless, overexpression of erbB3 has been generally considered as a poor prognostic factor in breast cancer patients [55]. This has been strongly supported by a recent study [56] showing that expression of erbB3 is associated with worse survival in a variety of human cancers of breast, colorectal, gastric, melanoma, ovarian, head and neck, pancreatic, and cervical; and the influence of erbB3 may be greater in the tumors with erbB2 overexpression. These data further emphasize the critical role of erbB2/erbB3 hetero-dimerization in cancer development.

### ErbB3 signaling in treatment resistance of cancer

Of the four erbB receptors, erbB3 is best suited to activate the PI-3 K/Akt signaling, because it has the most tyrosine residues on its C-terminal tail once being phosphorated, they are capable of binding to the p85 subunit of PI-3 K [12,57]. In fact, among all the erbB dimerization complexes, the erbB2/erbB3 hetero-dimer is the most biologically active and potent for activation of the PI-3 K/Akt signaling cascade [58,59]. Since PI-3 K/Akt signaling is the most important survival pathway in cell proliferation and its activation often leads to multidrug resistance in human cancers [60], it is understandable that one of the major biologic consequences of erbB3 activation is to cause treatment failure in cancer therapy [14].

Activation of erbB family members has been linked to tamoxifen resistance of estrogen receptor (ER) positive (ER+) breast cancers [61,62]. Cross-talk between ER and erbB2 or EGFR signaling promotes hormone-independent growth of breast cancer cells [63-65]. The importance of erbB3 in the development of a tamoxifen-resistant phenotype is emerging. A retrospective clinical study examining a large cohort of tamoxifen treated, ER + breast cancer patients found that the patients with co-expression of erbB2 and erbB3 were significantly more likely to relapse on tamoxifen [66]. Another study has shown that tamoxifen-sensitive MCF-7 cells transfected with a HRGß-2 cDNA become estrogen-independent and resistant to tamoxifen both *in vitro* and *in vivo*[65]. In prostate cancer, it is known that the erbB3/PI-3 K/Akt signaling plays a critical role in the development of castration-resistant prostate cancer (CRPC). While androgen withdrawal therapy (AWT) is an effective therapeutic intervention for recurrent prostate cancer, most patients ultimately develop resistance and progress to metastatic CRPC (mCRPC) [67]. It has been reported that elevated expression of erbB3 in CRPC leads to androgen receptor (AR) stabilization and activation of the PI-3 K/Akt signaling [17]. Thus, erbB3 receptor may serve as a useful biomarker for modulating tamoxifen sensitivity in luminal B (ER+, erbB2+) breast cancer and AWT in CRPC.

The EGFR- and/or erbB2-targeted therapies approved by FDA can be divided into two groups: 1) the blocking Abs, such as cetuximab (Erbitux) and panitumumab (Vectibix) against EGFR and trastuzumab (Herceptin), Pertuzumab (Perjeta), and T-DM1 (Kadcyla) against erbB2; 2) the small molecule tyrosine kinase inhibitors (TKIs), such as gefitinib (Iressa) and erlotinib (Tarceva) targeting EGFR and lapatinib (Tykerb/Tyverb) dual-targeting both EGFR and erbB2. All of these have been successfully used to treat a variety of human cancers. The majority of cancers develop resistance to these therapeutic Abs and/or inhibitors within one year. Numerous studies indicate that activation of erbB3 signaling is one of the major mechanisms of this resistance [68,69]. For example, a subset of colorectal cancer patients who exhibit either *de novo* or acquired resistance to cetuximab-based therapy has *erbB2* amplification or high levels of circulating HRG, which induces activation of erbB3 signaling [70]. The erbB3 signaling also contributes to gefitinib resistance in lung cancer-induced by gene amplification of *MET*[20]. A recent study showing that dual-targeting of EGFR and erbB3 is able to overcome acquired resistance to cetuximab and erlotinib further confirms the importance of erbB3 in the development of resistance to EGFR-targeted therapy [21]. In addition, transcriptional upregulation of *erbB3* has been shown to involve in resistance to RAF/MEK inhibitors in the treatment of melanoma and thyroid carcinomas [71,72]. It appears that different tumors utilize distinct mechanisms to upregulate erbB3. The RAF inhibitor PLX4720 in melanoma enhanced *erbB3* expression through the transcription factor, FOXD3 [71], whereas inhibition of RAF in thyroid cancers with vemurafenib (PLX4032) induced *erbB3* transcription via decreased promoter occupancy by the transcriptional repressors C-terminal binding protein 1 and 2 (CtBP1/2) [72]. Interestingly, the increased erbB3 in melanoma or thyroid cancers also depended upon erbB2 to activate the downstream signaling Akt [71] or MAPK [72]. Thus, in both studies, targeting of erbB2 with lapatinib was able to overcome the resistant phenotypes [71,72]. In light of the importance of enhanced erbB3 expression, we hypothesize that a novel strategy to inhibit erbB3 signaling or reduce erbB3 protein levels may exhibit an even better efficacy in combination with the RAF inhibitors.

Activation of the survival signaling - PI-3 K/Akt pathway by erbB3 (via interactions with another RTK, particularly erbB2) also gives rise to chemoresistance in cancer treatment. Docetaxel-based chemotherapy has been established as the standard of care for mCRPC. However, only half of the patients benefit from docetaxel. Of these, the majority will become resistance and eventually die of mCRPC [67,73]. Mechanistic studies suggest that activation of erbB3 signaling plays a vital role in the progression of mCRPC into docetaxel resistance [17]. Increased secretion of HRG has been found in a subset of ovarian cancers, and thereby stimulates ovarian cancer cell proliferation via erbB3/HRG autocrine loop [19]. Recent studies suggest that erbB3 signaling also contributes to chemoresistance in ovarian cancer, as the chemotherapeutic drug doxorubicin upregulates erbB3 ligands to activate the erbB3/PI-3 K/Akt signaling in ovarian cancer cells [74]. Thus, targeting of erbB3 may significantly sensitize ovarian tumors to the killing effects of platinum-based or other chemotherapy regimens [18]. Our early studies showed that co-expression of erbB2 and erbB3 in human breast cancer cell lines induced activation of PI-3 K/Akt signaling and was associated with an increased resistance to multiple chemotherapeutic agents, such as paclitaxel, doxorubicin, 5-fluorouracil, etoposide, and camptothecin [60].

**Figure 1:**
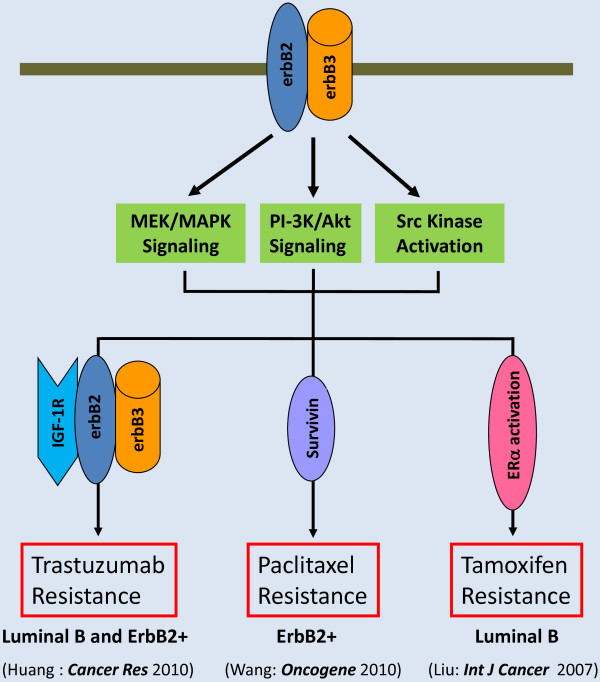
**ErbB3 interacts with erbB2 to activate signaling pathways leading to multi-drug resistance in breast cancer.** Hetero-dimerization of erbB2 and erbB3 is able to induce activation of multiple downstream signaling pathways. In both luminal B and erbB2+ subtypes of human breast cancer, erbB2/erbB3 association may recruit IGF-1R to form a trimeric complex activating PI-3 K/Akt signaling and Src kinase and resulting in trastuzumab resistance. In erbB2+ breast cancer, interaction between erbB2 and erbB3 upregulates Survivin via a PI-3 K/Akt-dependent mechanism, and thereby confers paclitaxel resistance. In luminal B breast cancer, the erbB2/erbB3 hetero-dimers modulate ERα phosphorylation (activation) mainly through MEK/MAPK and/or PI-3 K/Akt signaling pathways, and subsequently alter tamoxifen sensitivity. These data support the hypothesis that targeting of erbB3 will significantly enhance the efficacy of those commonly used therapeutics in the treatment of erbB2+ breast cancer.

In the last several years, our laboratory has focused on studying the unique biology of erbB3 receptor in the development of *erbB2* aberrant breast cancer. We have published a series of articles [31,32,75,76] indicating that estrogenic promotion of erbB2 kinase activity in mammary tumor cells requires erbB3, and the activation of erbB3 signaling plays an essential role in erbB2-mediated therapeutic resistance to tamoxifen, trastuzumab, and paclitaxel (Figure 1). We showed that specific knockdown of erbB3 by a siRNA abrogated erbB2-mediated tamoxifen resistance in breast cancer cells via enhanced apoptosis [31]. The molecular mechanism responsible for the increased sensitivity to tamoxifen upon erbB3 downregulation was due to decreased levels of phosphorylated Akt (P-Akt), altering the phosphorylation status of ERα. It is well-known that the PI-3 K/Akt signaling is associated with tamoxifen resistance and MCF-7 cells expressing a constitutively active Akt proliferate under reduced estrogen conditions and are resistant to tamoxifen-mediated growth inhibition [77-79]. Both erbB3 and IGF-1R-initiated signaling pathways contribute to trastuzumab resistance [80-82]. Nonetheless, the relationship between erbB3 and IGF-IR in trastuzumab resistance remains unclear. We recently discovered that the erbB2 receptor simultaneously interacted with erbB3 and IGF-1R to form a trimeric complex in trastuzumab-resistant breast cancer cells, and that it was the hetero-trimerization of erbB2/erbB3/IGF-1R, not the hetero-dimer of erbB2/erbB3 or IGF-1R/erbB2, that played a critical role in the breast cancer cells resistant to trastuzumab [75]. Further studies showed that specific knockdown of either erbB3 or IGF-1R was able to reverse trastuzumab resistance, and significantly enhanced trastuzumab-mediated growth inhibitory effects on the otherwise resistant cells. For the downstream signaling, specific knockdown of erbB3 decreased the levels of both P-Akt and P-Src, whereas IGF-1R knockdown only gave rise to reduction of P-Src [75]. Our data suggest that erbB3 and IGF-1R initiate different signaling pathways contributing to trastuzumab resistance - erbB3 activates both PI-3 K/Akt signaling and Src kinase, whereas IGF-1R mainly elicits Src activation. In identifying the key downstream mediators through which erbB3 contributes to chemoresistance, we found that elevated expression of erbB3 conferred paclitaxel resistance in erbB2+ breast cancer cells via PI-3 K/Akt-dependent upregulation of Survivin, a critical inhibitor of apoptosis [76]. Survivin is selectively expressed in a variety of human malignancies and its expression positively correlates with poor prognosis, tumor recurrence and therapeutic resistance [83]. Thus, novel strategies targeting Survivin, such as antisense oligonucleotide and pharmacological inhibitors may significantly enhance chemotherapeutic efficacy and are currently under clinical trials for cancer treatment [83-85]. Nonetheless, the precise mechanism by which erbB3 signaling specifically upregulates Survivin, but not the functionally related molecules Mcl-1 and Bcl-xL in erbB2+ breast cancer cells [76] remains unknown.

### Strategies to inhibit erbB3 signaling for cancer therapy

**Figure 2:**
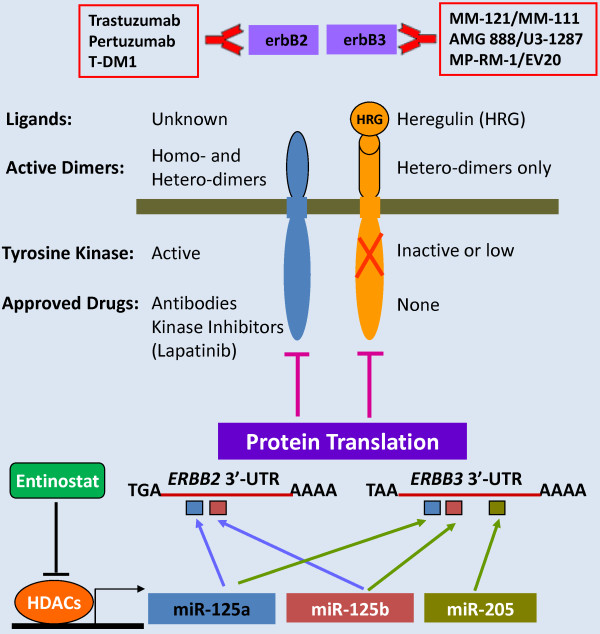
**Current and novel therapeutic strategies targeting of erbB2 and/or erbB3 receptors for cancer therapy.** Several erbB2-targeted therapies (trastuzumab, pertuzumab, T-DM1, and lapatinib) have being used in clinic, whereas no erbB3-targeted therapy has been approved for cancer treatment. Blocking Abs, such as MM-121, MM-111, AMG 888/U3-1287, and MP-RM-1/EV20, are the only agents being tested in early clinical and/or preclinical investigations. Our recent data show that entinostat, a class I HDAC inhibitor selectively downregulates erbB2/erbB3 via induction of specific miRNAs, miR-125a, miR-125b, and miR-205 in erbB2+ breast cancer cells. Further characterization demonstrates that these “sister” miRNAs (share common targets) act in concert to inhibit erbB2/erbB3 protein translation. Thus, the novel strategy, like cooperative miRNA targeting of erbB2/erbB3 may represent a new approach for cancer therapy.

It is clear that there is overwhelming evidence to support the importance of erbB3 signaling in cancer progression, particularly in therapeutic resistance followed by tumor recurrence [14]. Concomitant inhibition of erbB3 is thought to be required to overcome the resistance and effectively treat cancer patients. Indeed, advances have been made in developing erbB3-targeted therapy [86], and several anti-erbB3 monoclonal Abs exhibit efficacy *in vivo* and show promise as novel cancer therapeutics [87,88]. Both erbB3-specific inhibitors and pan-erbB inhibitors that simultaneously inhibit erbB3 and other family members have been developed, and a number of them are in early clinical development. A computational model was used to explore the optimal way to therapeutically inhibit the erbB3 signaling-induced by combinatorial ligands [23]. This study revealed a dominant role of erbB3 in Akt activation and suggested that targeting this key node of the erbB signaling network might result in therapeutic benefit to cancer patients. However, a principal challenge to target erbB3 is that, unlike other erbB family members, the erbB3 receptor lacks or possesses much lower intrinsic kinase activity [10,11], suggesting that its function cannot be inhibited by a small molecule inhibitor (Figure 2). Thus, targeting of erbB3 with a blocking Ab is the only strategy currently under preclinical investigation [22,23] and clinical evaluation in patients with advanced solid tumors (http://www.clinicaltrials.gov). Recent studies have identified bispecific Abs dual-targeting of EGFR/erbB3 [21] or erbB2/erbB3 [25], that exert potent antitumor activities in laboratory research and are now in clinical testing [86]. In addition, the erbB3 inhibitors based on a novel biologic scaffold termed a surrobody have been developed and show inhibitory effects on tumor cell proliferation *in vitro* and *in vivo*[89].

MM-121 is a fully humanized anti-erbB3 monoclonal IgG2 Ab. It inhibits ligand-induced dimerization of erbB3 and erbB2 and subsequently inactivates the downstream signaling. MM-121 has been shown to exert antitumor activity in preclinical models of various human cancers [22,23]. At present, clinical trials are recruiting patients with advanced solid tumors for phase I or II studies of MM-121 in combination with chemotherapy or other erbB inhibitors. However, whether MM-121 has the therapeutic potential to overcome resistance to trastuzumab or chemotherapy, like paclitaxel in erbB2+ breast cancer cells remains unclear. We have recently tested the hypothesis that MM-121 abrogates erbB3 signaling-mediated resistance to trastuzumab and paclitaxel in erbB2+ breast cancer cells via inactivation of erbB3 and its downstream PI-3 K/Akt signaling. Our data showed that MM-121 reduced the expression of Survivin, overcame paclitaxel resistance, and significantly enhanced paclitaxel-induced apoptosis in the otherwise resistant breast cancer cell lines [90]. We also found that treatment of erbB2+ breast cancer cell lines refractory to trastuzumab with MM-121 resulted in a dramatic inhibition of PI-3 K/Akt signaling. MM-121 significantly enhanced trastuzumab-induced growth inhibition in erbB2+ breast cancer cell lines, and was able to overcome trastuzumab resistance [91]. While MM-121 in combination with trastuzumab mainly inhibited proliferation via cell cycle G1 arrest *in vitro*, their combinatorial *in vivo* antitumor activity could be attributed to induction of both growth inhibition and apoptosis [91]. Our data strongly support the development of clinical studies to evaluate the efficacy of MM-121 in combination with trastuzumab or paclitaxel in therapeutically resistant erbB2+ breast cancer patients. MM-111 is a bispecific antibody, dual-targeting erbB2/erbB3 and inhibiting the downstream signaling, like PI-3 K/Akt pathway [25]. The safety and clinical activity of MM-111 is now being tested in several phase I clinical trials. Another erbB3-targeted drug, U3-1287/AMG-888, is the first fully humanized anti-erbB3 monoclonal Ab and currently under clinical studies in patients with advanced solid tumors as well. This Ab inhibits proximal and distal erbB signaling and induces rapid internalization of erbB3 [92]. AMG-888 shows growth inhibitory effects on multiple cancer cell lines (breast, lung, colorectal) that are resistant to other erbB inhibitors [92]. It also significantly decreases colony formation in pancreatic cancer cells and tumor growth in pancreatic cancer, NSCLC, and colorectal cancer xenograft models [17]. Recently, a new anti-erbB3 Ab (MP-RM-1) and its humanized version (named EV20) both have been shown to exhibit antitumor activity against various cancer types *in vitro* and *in vivo*[93,94]. Because this Ab has the ability to inhibit both ligand-dependent and -independent activation of erbB3 [93,94], we speculate that EV20 may have a much broader effect on blocking erbB3 signaling than those Abs (like MM-121) which only prevent ligand-induced activation of erbB3, and thus exert more potent activity to overcome drug resistance in cancer therapy.

Most erbB3-targeted therapies under development aim to inhibit erbB3 signaling by targeting the extracellular domain of the receptor. Novel approaches targeting of erbB3 have been proposed. One of these, EZN-3920 (Enzon Pharmaceuticals, Inc., Piscataway, NJ) is a high-affinity, locked nucleic acid (LNA) antisense oligonucleotide. It specifically downregulates erbB3 and demonstrates significant antitumor activity in mouse xenograft models of breast and lung cancer cell lines [95]. Clinical testing of EZN-3920’s activity in cancer patients is not initiated yet. We reported that the specific class I HDAC inhibitor entinostat (also known as MS-275 or SNDX-275, Syndax Pharmaceuticals, Inc., Waltham, MA) selectively downregulated erbB2/erbB3 receptors and induced apoptosis in erbB2+ breast cancer cells [96]. It appeared that entinostat reduced erbB2/erbB3 through a transcription-independent mechanism, as it had no effect on mRNA levels of *erbB2/erbB3*. Further characterization revealed that entinostat upregulated three *erbB2*/*erbB3*-targeting miRNAs (miR-125a, miR-125b, and miR-205) which acted in concert to inhibit erbB2/erbB3 translation [97]. Thus, miRNA-mediated epigenetic regulation may represent a new mechanism inactivating erbB2/erbB3. We hypothesize that targeting of erbB3 by “sister” miRNAs [98] via functional cooperation may be developed as a novel therapeutic strategy against erbB3 (Figure 2). Detailed analysis is warranted to test this innovative idea *in vitro* and *in vivo*.

### Conclusions and future directions

Research on erbB receptors has long been focused on dysregulation of tyrosine kinase activity of EGFR and erbB2 in human cancers. Recently, the role of erbB3 as an obligate partner and in primary and acquired resistance to cancer therapeutics has attracted considerable attention. Increased awareness of erbB3 function in cancer progression, particularly tumor recurrence following drug resistance has several implications for future directions of investigation. ErbB3 may be considered as a valuable biomarker to predict the efficacy of EGFR- and/or erbB2-targeted therapy in the treatment of NSCLC and erbB2+ breast cancer, respectively. Therapeutic targeting of erbB3 has been shown to be an effective way to conquer drug resistance and significantly enhance the antitumor activity of hormonal therapy, targeted therapy, chemotherapy, and radiotherapy. Although numerous studies have dramatically improved our understanding of the biologic features of erbB3 receptor in cancer biology, and advances have been achieved in developing Abs against erbB3 with therapeutic potential, a number of critical questions still exist. First, activation of erbB3 signaling not only confers drug resistance in cancer treatment, but also promotes tumor metastasis [99-103]. The critical downstream mediators that are responsible for erbB3 signaling-induced cancer metastasis remain unclear. We found that PI-3 K/Akt-dependent upregulation of Survivin played a vital role in erbB3-mediated paclitaxel resistance in erbB2+ breast cancer cells, and specific knockdown of Survivin abrogated the resistance [76]. However, it is unknown whether the increased Survivin may lead to resistance to all therapeutic agents. Identifying other mediators of erbB3 signaling may provide additional opportunities to develop novel strategies revoking drug resistance and tumor metastasis. Second, when considered individually, both erbB2 and erbB3 have defects in that erbB2 has no known ligand and erbB3 has impaired kinase activity. These two receptors rely on each other to elicit the most biologically active and potent activation of PI-3 K/Akt signaling [58,59]. Nonetheless, the molecular basis through which tumor cells often co-express erbB2 and erbB3 remains elusive. It is not clear whether same mechanisms are utilized to simultaneously upregulate both erbB2 and erbB3, or whether tumor cells first overexpress one receptor which subsequently enhances expression of the other receptor. Third, targeting of erbB3 with a blocking Ab is active in both preclinical and clinical studies. However, tumor cells may eventually develop resistance to the anti-erbB3 Abs, since the Abs like EGFR/erbB2-targeted therapies just inhibit signaling without altering expression of the erbB receptors. We believe that new strategies/agents that aim to reduce erbB3 protein levels, such as the antisense oligonucleotide EZN-3920 [95], the HDAC inhibitor entinostat [96], and the cooperative “sister” miRNAs [97,98] may hold special antitumor activity as the tumor cells won’t have opportunities to develop resistance. Finally, HRG is vital to induce activation of erbB3 signaling. Aberrant production and/or maturation of HRG will affect tumor cell survival and proliferation. Thus, studies on dysregulation of HRG in cancers may improve our understanding of the ligand’s biological function in erbB3-mediated tumor initiation and progression, and facilitate the development of novel strategy for cancer therapy. It is conceivable to hypothesize that novel approaches against HRG, such as neutralization Abs, may exert similar anti-tumor efficacy as therapeutic targeting of erbB3.

## Abbreviations

FDA: Food and Drug Administration; RTK: Receptor tyrosine kinase; ER: Estrogen receptor; PR: Progesterone receptor; AR: Androgen receptor; EGFR: Epidermal growth factor receptor; HRG: Heregulin; IGF-I: Insulin-like growth factor-I; IGF-1R: IGF-I receptor; PTEN: Phosphatase and tensin homolog; PI-3 K: Phosphoinositide 3-kinase; MAPK: Mitogen-activated protein kinase; MEK: MAPK kinase; AWT: Androgen withdrawal therapy; Ab: Antibody; TKI: Tyrosine kinase inhibitor; CRPC: Castration-resistant prostate cancer; mCRPC: Metastatic CRPC; NSCLC: Non-small cell lung cancers; IHC: Immunohistochemistry; CtBP1/2: C-terminal binding protein 1 and 2.

## Competing interests

The authors declare that they have no competing interests.

## Authors’ contributions

JM, HL, JH, and BL drafted and finalized the manuscript. All authors read and approved the final manuscript.
